# Impact of lighting color and duration on productive performance and Newcastle disease vaccination efficiency in broiler chickens

**DOI:** 10.14202/vetworld.2019.1052-1059

**Published:** 2019-07-17

**Authors:** Essam S. Soliman, Rania A. Hassan

**Affiliations:** 1 Department of Animal Hygiene, Zoonosis, and Animal Behavior, Faculty of Veterinary Medicine, Suez Canal University, Ismailia 41522, Egypt; 2 Department of Animal Wealth Development, Animal Production Division, Faculty of Veterinary Medicine, Suez Canal University, Ismailia 41522, Egypt

**Keywords:** broilers, light-emitting diode colors, metabolism, Newcastle vaccine, productive performance

## Abstract

**Background and Aim:**

Manipulating lighting colors and regimens is considered an effective mean for improving broiler productivity. The influence of red, blue, and white light-emitting diode (LED) was investigated using three different regimens of lighting and darkness; continuous 23 h light (L):1 h dark (D), continuous 18 h L:6 h D, and intermittent 16 h L:8 h D hours on the performance, carcass weight (CW), feed and water intake (WI), serum glucose (GLUCO), triglycerides (TG), and cholesterol (TC), intestinal bacterial load, growth and metabolic hormones, and efficiency of Newcastle disease (ND) vaccine.

**Materials and Methods:**

A total of 252 1-day-old Ross broilers on deep litter were divided into nine groups. The 1^st^, 4^th^, and 7^th^ groups were exposed to continuous 23L:1D, the 2^nd^, 5^th^, and 8^th^ groups were exposed to continuous 18L:6D, and the 3^rd^, 6^th^, and 9^th^ groups were exposed to intermittent 16L:8D (4L:2D, 4 times) lighting regimen using red, blue, and white LED lights, respectively. A total of 1350 samples (225 sera, 225 swabs, and 900 organ samples) were collected.

**Results:**

Blue LED group revealed a highly significant increase (p<0.01) in live body weight, body weight gain, performance index, CW, spleen, heart, and liver weights, and anti-ND antibody titer, as well as a highly significant decline (p<0.01) of feed intake, WI, GLUCO, TG, TC, growth hormone, insulin, tri-iodothyronine (T3), tetra-iodothyronine (T4), total bacterial count (TBC), and total *Enterobacteriaceae* count compared to red and white LED lights in all tested lighting regimens. Continuous 23L:1D and 18L:6D regimens were significantly (p<0.01) superior to intermittent 16L:8D in their influence on the performance, CW, biochemistry, hormonal profile, and bacterial load.

**Conclusion:**

The blue LED light associated with continuous 18L:6D or 23L:1D h regimen is highly recommended in broiler houses for their enhancing the productive performance, growth, and immunity.

## Introduction

Light is a key microclimatic factor that hits broiler skull at the retinal receptors and traveling through neurons to the pineal gland, stimulating pineal gland, and hypothalamus regulating functions including metabolism and reproduction [1]. Light is known for its influence on growth performance, immunity levels, metabolism, behavior, and bird activity [2,3]. The eye and visual cortex represent anatomically a large proportion in the broiler brain. Eyes are important in realizing the extension of the landscape, recognizing features of other birds, and providing a probable response to humans. Broilers perceive light signals from the surrounding microclimate through photoreceptors that consist of one rod and six cones. Photosensitive pigments in retinal rods and cones relay these light signals to central neurons, where signals are integrated into an image [4].

Light color (wavelength) is an important component of the physical light environment that affects broiler growth, performance, and welfare. Long wavelengths are known for higher penetration power compared to short wavelengths [5]. Artificial lighting has been used in modern poultry production to stimulate productive performance. The light-emitting diode (LED) provides an approximation of daylight than the spectral gaps of other lighting sources [6]. Researches conducted on light color suggested the blue, green lights [7,8], and white lights [9] to enhance broiler growth, production, and act on increasing myofiber growth through effective stimulation of testosterone.

Photoperiod is defined as the length of light duration, while scotoperiod is the length of dark hours. Lighting regimens in the poultry industry have been fluctuated between continuous and intermittent lighting according to their effectiveness on welfare and productive performance of broilers [10,11]. Continuous lighting systems combined with *ad libitum* feeding adversely affected broilers performance compared to restricted lighting’ and feeding regimen [12]. Modern poultry production was associated with intensification and genetic selection for rapidly growing broilers, that is, why many researchers investigated and noted that a longer photoperiod and a shortened scotoperiod contributed to increased livability and feed intake (FI), and thus increased weight gain (WG) in broilers [13].

This study aimed to investigate the influence of manipulating lighting color as red, blue, and white LED and duration (continuous 23L:1D, continuous 18L:6D, and intermittent 16L:8D hours) on productive performance, feed and water intake (WI), carcass weight (CW), immune organs’ (spleen and bursa) and edible organs’ (liver and heart) weights, biochemical parameters (glucose [GLUCO], triglycerides [TG], and total cholesterol [TC]), intestinal bacterial load, growth and metabolic hormones, and efficiency of Newcastle virus vaccine.

## Materials and Methods

### Ethical approval

The protocol of the present study was approved by the Scientific Research Ethics Committee of Faculty of Veterinary Medicine, Suez Canal University, Ismailia, Egypt, (2018061).

### Experimental plan and housing management

A total of 252 1-day-old Ross broilers were purchased from El-Frog Co-Ismailia, divided into nine groups of 28 broilers each (four replicates, each one seven chicks), and housed on a deep litter system. The floor of the building was treated with superphosphate 0.5 g/m^2^, according to Soliman *et al*. [14] to absorb moisture, minimize ammonia evaporation, and reduce microbial survival.

The building was divided interiorly using dark brown blackout curtains into nine sections, each of 3 m^2^ and used for one group and its assigned lighting program. Each section was provided with a V-shaped window that was covered with dark blackout to prevent their interference with the lighting program and a ceiling fan to encourage air exchange and stimulate stack effect. An automated LED lamp of 18 watt, 1750 lumen, and monochromic (red, blue, and white) was supplied in each section and adjusted by timer according to the recommended light (L)/dark (D) hours for each group. The experiment was designed to past for 40 days, during which mortalities, temperature, and relative humidity were monitored daily.

### Broiler microclimate

Broilers were brooded at 35°C and decreased by 3°C weekly until achieving 21-24°C by the 3^rd^ week. Broilers were supplied with standard corn-soybean ration, as shown in Table-1, to satisfy the basic requirements as recommended by the National Research Council [15]. In addition, they were given *ad libitum* access to water. Broilers were vaccinated using drinking water mass vaccination with live attenuated virus of IB-H120 ≥10^3.5^ against infectious bronchitis at day 6, initial and booster doses of live attenuated virus of VMG91 ≥10^3.0^ against infectious bursal disease at days 14 and 21, respectively, and with initial and booster doses of live lentogenic Newcastle disease (ND) virus of Lasota ≥10^6.0^ against (ND) virus at days 18 and 28, respectively.

**Table 1:** Feed ingredients and nutrient contents of standard soybean ration at different growth stages of the experiment.

Ingredients %	Starter 1:14days	Grower 15:40days
Corn	50.00	56.50
Soybean meal	39.00	33.50
Fish meal	3.00	2.50
Calcium carbonate	2.00	2.00
Monocalcium phosphate	2.00	2.00
DL-methionine	0.50	0.50
L-lysine	0.50	0.50
Vitamin mix	2.50	2.50
Common salt	0.50	0.50
Energy	2990 Kcal/kg	3200 Kcal/kg
Protein	22%	21%
Fat	3.5%	5.5%
Crude fiber	3.4%	3.7%

### Lighting colors and durations regimens

Broilers of Group 1 (G1), Group 2 (G2), and Group 3 (G3) were exposed to red LED light, Group 4 (G4), Group 5 (G5), and Group 6 (G6) were exposed to blue LED light, and Group 7 (G7), Group 8 (G8), and Group 9 (G9) were exposed to white LED light. Light (L) to dark (D) hours were adjusted automatically by timer in each group as following: G1, G4, and G7 were exposed to continuous lighting using 23L:1D hours regimen, G2, G5, and G8 were exposed to continuous lighting using 18L:6D hours regimen, and G3, G6, and G9 were exposed to intermittent lighting using 16L:8D hours regimen (4L: 2D, 4 times).

### Performance indices (PI)

FI/g of each bird was calculated from the total amount consumed based on each group’s intensity (the absolute number of birds in each group). Live body weight (LBW/g) was estimated by weighing at least 26 birds per group every week, the number was calculated by simple random sampling design as recommended by Thrusfield [16] with an expected error 5%:

n=1.96^2^ P_exp_(1-P_exp_)/d^2^

Where n=required sample size, P_exp_=Expected prevalence, d=Desired absolute precision. Performance indices including body WG (BWG/g), feed conversion ratio (FCR), and performance index (PI) were calculated as recommended by Soliman and Hassan [17]. Mortalities were calculated as a proportion between the numbers of succumbed birds from the total population at risk.

### Sampling

A total of 1350 samples (225 sera, 225 intestinal swabs, and 900 organ samples including edible organs as liver and heart, and immune organs as spleen and bursa) were collected by the end of the experiment (40 days). Blood samples were collected, held at 37°C for 2 h, and centrifuged at 3500 rpm for 15 min. Clear sera were dispensed into Eppendorf tubes, tested for blood sugars, and stored at −20°C for biochemical, hormonal, and immunological analysis [18]. Birds were slaughtered after blood sampling; carcasses were weighed (total CW and expressed by CW/g), liver, heart, spleen, and bursa were removed, weighed, and expressed as g/kg. Swab samples were collected from the intestine, added to 9 mL buffered peptone water, preserved in an icebox and transferred to the laboratory for bacteriological evaluation.

### Biochemical and hormonal profile

Sera were examined for GLUCO mg/dl, TG mg/dl, and TC mg/dl calorimetrically using Roche COBAS INTEGRA^®^ 400 Plus Analyzer. Growth hormone (GH ng/mL), insulin (µIU/mL), tri-iodothyronine (T3 ng/mL), tetra-iodothyronine (T4 ng/mL), and Newcastle virus vaccine titer (ND mg/dl) were measured using Roche ELECSYS 1010 Immunoassay Analyzer [19].

### Bacteriological examination

Intestinal swabs were subjected to ten-fold serial dilutions up to 10^−6^, as recommended by APHA [20]. Total bacterial count (TBC) onto standard plate count agar and total *Enterobacteriaceae* count (TEC) onto eosin methylene blue agar at 37°C for 24-48 h were performed using a drop plate technique [21,22]. Plates were counted using Darkfield Colony Counter [23].

### Statistical analysis

Statistical analysis was carried out using a Statistical Package for the Social Sciences version 20 (IBM SPSS Statistics 20) [24]. The obtained data were analyzed statistically using a multifactorial analysis of variance. Bacterial counts were transferred into logarithmic counts using Microsoft Excel.

## Results

Crude mortality rates revealed a total of 8.7% (22 out of 252 birds), including 35.71% (10 out of 28 birds), 17.85% (5 out of 28 birds), 10.71% (3 out of 28 birds), and 14.28% (4 out of 28 birds) mortality rates in broilers raised in continuous 23L:1D hours regimen of red, intermittent 16L:8D hours regimen of red, intermittent 16L:8D hours regimen of blue, and intermittent 16L:8D hours regimen of white light system, respectively.

Performance indices, in Table-2, revealed a highly significant increase (p<0.01) of BWG and PI among broilers reared in continuous 23L:1D and 18L:6D hours regimens of blue light compared to intermittent lighting regimen. Meanwhile, the lowest significant (p<0.01) FCR was recorded in continuous 23L:1D hours regimen of blue light.

**Table 2:** Performance indices (Mean±SE) in broilers exposed to lighting regimens using different lighting colors and durations.

Light color	Photo-period L: Dhour	BWG/g	FCR %	PI	WI/mL	FI/g	WI: FI ratio
Red		253.0^c^±13.0	2.18^a^±0.07	3.3^c^±0.21	530.8^a^±28.1	206.2^a^±16.4	0.35^a^±0.012
Blue		391.8^a^±21.7	1.14^c^±0.04	9.4^a^±0.78	433.9^b^±23.1	163.8^a^±13.0	0.34^a^±0.013
White		329.5^b^±15.5	1.43^b^±0.06	6.3^b^±0.40	466.5^ab^±24.6	190.7^a^±14.8	0.37^a^±0.013
p-value		0.000	0.002	0.000	0.026	0.056	0.310

**Light’s color X lighting program**							

Red							
	C-23:1	270.7^a^±24.7	2.16^b^±0.14	3.6^a^±0.43	556.3^a^±50.1	224.9^a^±30.5	0.37^a^±0.021
	C-18:6	258.5^b^±22.4	2.10^c^±0.11	3.4^b^±0.37	530.2^ab^±49.6	205.3^b^±28.6	0.35^a^±0.020
	I-16:8	229.8^c^±20.6	2.30^a^±0.13	2.9^c^±0.29	505.9^b^±47.6	188.2^c^±26.9	0.33^a^±0.021
Blue							
	C-23:1	415.6^a^±38.9	1.12^c^±0.07	9.6^a^±1.31	464.1^a^±44.2	170.1^a^±23.3	0.33^a^±0.018
	C-18:6	384.4^b^±37.1	1.15^b^±0.08	9.3^b^±1.42	425.0^ab^±38.1	163.0^b^±22.7	0.35^a^±0.025
	I-16:8	375.4^c^±38.0	1.16^a^±0.09	9.1^c^±1.40	412.6^b^±38.2	158.5^c^±22.6	0.34^a^±0.025
White							
	C-23:1	352.7^a^±26.4	1.39^c^±0.10	6.8^a^±0.67	497.1^a^±46.3	201.5^a^±26.5	0.37^a^±0.019
	C-18:6	325.0^b^±28.2	1.45^a^±0.11	6.1^b^±0.74	462.8^ab^±42.2	189.4^b^±26.2	0.37^a^±0.023
	I-16:8	310.7^c^±26.2	1.44^b^±0.12	5.9^c^±0.68	439.5^b^±40.3	181.3^c^±25.3	0.37^a^±0.024
p-value		0.009	0.003	0.007	0.000	0.024	0.860

Means carrying different superscripts in the same column are significantly different at (p≤0.05) or highly significantly different at p<0.01. Means carrying the same superscripts in the same column are non-significantly different at p<0.05. L=Light hours, D=Dark hours, C=Continuous, I=Intermittent, BWG=Body weight gain, FCR=Feed conversion ratio, PI=Performance index, WI=Water intake, FI=Feed intake, WI: FI=Water intake to Feed intake ratio, SE=Standard error

WI revealed a highly significant increase (p<0.01), as shown in Table-2, between broilers raised in continuous 23L:1D hours regimen and those raised in intermittent 16L:8D hours of red light with no significant differences between neither of them and continuous 18L:6D. FI revealed no significant difference in the overall comparison between all broiler groups raised under different systems. On calculating WI/FI ratio (Table-2), no significant differences were revealed between the nine lighting regimens.

Continuous 23L:1D and 18L:6D hours regimens of blue light revealed a highly significant increase (p<0.01) of LBW, CW, spleen, and heart weights compared to other lighting colors and regimens (Table-3). Furthermore, liver and bursa revealed a highly significant increase (p<0.01) in broilers raised in continuous 23L:1D and 18L:6D hours regimens of blue and red lights with no significant differences in between (Table-3).

**Table 3:** Terminal marketing body weight and carcass quality characteristics (Mean±SE) in broilers exposed to lighting regimens using different lighting colors and durations.

Light color	Photo-period L: Dhour	LBW/g	CW/g	Organs/CW ratio			

				Liver %	Spleen %	Heart %	Bursa %
Red		1300^c^±12.5	1007^c^±11.6	1.90^a^±0.04	0.05^c^±0.00	0.34^c^±0.01	0.12^a^±0.00
Blue		2028^a^±10.9	1749^a^±9.1	1.98^a^±0.04	0.09^a^±0.00	0.47^a^±0.02	0.06^b^±0.00
White		1703^b^±12.7	1418^b^±10.3	1.68^b^±0.04	0.08^b^±0.01	0.40^b^±0.02	0.05^b^±0.00
p-value		0.002	0.000	0.000	0.000	0.000	0.096

**Light’s color X lighting program**							

Red							
	C-23:1	1382^a^±17.3	1086^a^±11.4	2.0^a^±0.07	0.06^a^±0.00	0.39^a^±0.01	0.13^a^±0.01
	C-18:6	1348^b^±5.3	1059^a^±7.2	1.7^c^±0.06	0.05^b^±0.00	0.31^b^±0.01	0.12^b^±0.01
	I-16:8	1171^c^±7.7	876^b^±4.9	1.9^b^±0.08	0.05^b^±0.00	0.30^b^±0.02	0.11^c^±0.01
Blue							
	C-23:1	2150^a^±9.7	1896^a^±10.2	2.2^a^±0.05	0.11^a^±0.01	0.57^a^±0.02	0.07^a^±0.00
	C-18:6	1990^b^±5.3	1712^b^±6.2	2.0^b^±0.05	0.09^b^±0.01	0.47^b^±0.03	0.06^a^±0.01
	I-16:8	1944^c^±3.8	1638^c^±4.1	1.6^c^±0.05	0.08^c^±0.01	0.37^c^±0.03	0.05^a^±0.01
White							
	C-23:1	1847^a^±10.9	1568^a^±9.8	1.8^a^±0.06	0.08^a^±0.01	0.45^a^±0.03	0.06^a^±0.01
	C-18:6	1657^b^±5.1	1376^b^±4.9	1.7^b^±0.07	0.07^b^±0.01	0.39^b^±0.03	0.05^a^±0.01
	I-16:8	1606^c^±3.6	1310^b^±3.2	1.5^c^±0.07	0.08^a^±0.01	0.35^c^±0.04	0.04^a^±0.01
p-value		0.000	0.000	0.002	0.068	0.005	0.006

Means carrying different superscripts in the same column are significantly different at p≤0.05 or highly significantly different at p<0.01. Means carrying the same superscripts in the same column are non-significantly different at p<0.05. L=Light hours, D=Dark hours, C=Continuous, I=Intermittent, LBW=Live body weight, CW=Carcass weight, SE=Standard error

GLUCO, TG, and TC revealed, in Table-4, the highest significance (p<0.01) in red light. Meanwhile, the lowest significance (p<0.01) of TG and TC was recorded in blue and white lights. A synchronized highly significant decline (p<0.01) of GLUCO, TG, and TC was recorded, as shown in Table-4, in continuous 23L:1D, continuous 18L:6D, and intermittent 16L:8D hours regimens, respectively.

**Table 4:** Biochemical profile (Mean±SE) in broilers exposed to lighting regimens using different lighting colors and durations.

Light color	Photo-period L: Dhour	GLUCO mg/dl	TG mg/dl	TC mg/dl
Red		237.1^a^±5.03	235.7^a^±2.42	317.1^a^±6.92
Blue		157.8^b^±4.84	108.3^c^±1.90	148.7^c^±4.56
White		118.0^c^±3.87	144.0^b^±2.24	247.7^b^±5.55
p-value		0.000	0.000	0.000

**Light’s color X lighting program**				
Red				

	C-23:1	184.2^c^±2.12	218.4^c^±2.51	246.8^c^±2.43
	C-18:6	240.1^b^±2.16	230.2^b^±1.56	314.7^b^±3.11
	I-16:8	287.2^a^±2.03	258.4^a^±1.09	389.7^a^±2.55
Blue				
	C-23:1	114.7^c^±2.15	88.2^c^±0.91	108.3^c^±2.71
	C-18:6	147.1^b^±2.18	110.1^b^±0.56	140.4^b^±2.11
	I-16:8	211.7^a^±1.55	126.6^a^±0.87	197.5^a^±1.87
White				
	C-23:1	79.7^c^±1.49	121.8^c^±1.69	184.7^c^±1.19
	C-18:6	115.0^b^±1.08	146.1^b^±1.52	259.6^b^±1.11
	I-16:8	159.1^a^±1.14	164.2^a^±1.22	299.0^a^±0.98
p-value		0.000	0.002	0.000

Means carrying different superscripts in the same column are significantly different at p≤0.05 or highly significantly different at p<0.01. Means carrying the same superscripts in the same column are non-significantly different at p<0.05. L=Light hours, D=Dark hours, C=Continuous, I=Intermittent, GLUCO=Glucose, TG=Triglycerides, TC=Total cholesterol, SE=Standard error

GH, insulin, T3, and T4 hormones, in Table-5, revealed a highly significant increase (p<0.01) in broilers reared in red light compared to those reared in blue and white lights. Meanwhile, ND titer revealed a highly significant increase (p<0.01) in blue, white, and red lights, respectively. GH and ND vaccine titer revealed a highly significant increase (p<0.01) in continuous 23L:1D, continuous 18L:6D, and intermittent 16L:8D hours regimens, respectively, although that insulin, T3, and T4 hormones revealed a highly significant decline (p<0.01) in the same lighting regimens (Table-5).

**Table 5:** Hormonal profile and Newcastle virus vaccine titer (Mean±SE) in broilers exposed to lighting regimens using different lighting colors and durations.

Light color	Photo-period L: Dhour	GH ng/mL	Insulin µIU/mL	T3 ng/mL	T4 ng/mL	ND Titer mg/dl
Red		109.8^a^±0.78	20.5^a^±0.16	202.2^a^±1.46	12.2^a^±0.11	212.4^c^±8.15
Blue		19.4^b^±1.76	15.3^b^±0.30	107.5^c^±3.04	5.7^c^±0.21	420.7^a^±8.10
White		18.5^c^±0.23	7.2^c^±0.18	150.7^b^±2.32	7.5^b^±0.19	307.1^b^±4.35
p-value		0.000	0.000	0.002	0.005	0.001

**Light’s color X lighting program**						

Red						
	C-23:1	117.5^a^±0.72	18.7^c^±0.09	189.1^c^±1.25	11.3^c^±0.13	286.1^a^±3.93
	C-18:6	108.5^b^±0.68	20.8^b^±0.08	201.1^b^±1.33	12.2^b^±0.09	228.7^b^±3.36
	I-16:8	103.5^c^±0.55	22.0^a^±0.09	216.2^a^±1.19	13.0^a^±0.11	123.2^c^±2.98
Blue						
	C-23:1	40.7^a^±0.66	12.0^c^±0.12	77.6^c^±0.74	3.6^c^±0.07	497.7^a^±4.04
	C-18:6	9.71^b^±0.11	15.8^b^±0.11	103.9^b^±0.82	5.5^b^±0.08	429.5^b^±3.56
	I-16:8	8.0^c^±0.12	18.1^a^±0.09	140.9^a^±0.77	8.1^a^±0.07	334.6^c^±4.12
White						
	C-23:1	20.7^a^±0.17	5.4^c^±0.08	126.0^c^±1.32	5.6^c^±0.09	352.1^a^±2.94
	C-18:6	18.6^b^±0.18	7.2^b^±0.07	154.0^b^±0.99	7.4^b^±0.08	301.3^b^±2.22
	I-16:8	16.3^c^±0.23	9.6^a^±0.08	172.1^a^±1.41	9.5^a^±0.08	268.4^c^±2.54
p-value		0.000	0.000	0.000	0.002	0.003

Means carrying different superscripts in the same column are significantly different at p≤0.05 or highly significantly different at p<0.01. Means carrying the same superscripts in the same column are non-significantly different at p<0.05. L=Light hours, D=Dark hours, C=Continuous, I=Intermittent, GH=Growth hormone, T3=Tri-iodothyronine, T4=Tetra-iodothyronine, ND=Newcastle, SE=Standard error

Log TBC and TEC showed, in Table-6, a highly significant decline (p<0.01) in continuous 23L:1D, continuous 18L:6D, and intermittent 16L:8D hours regimens of blue, white, and red lighting regimens, respectively.

**Table 6:** Logarithmic bacterial counts (total bacterial and *Enterobacteriaceae* counts Mean±SE) in broilers exposed to lighting regimens using different lighting colors and durations.

Light color	Photo-period L: Dhour	Log. TBC CFU/mL	Log. TEC CFU/mL
Red		5.25^a^±0.022	2.22^a^±0.022
Blue		3.81^c^±0.013	0.85^c^±0.044
White		4.37^b^±0.011	1.31^b^±0.025
p-value		0.000	0.000

**Light’s color X lighting program**			

Red			
	C-23:1	5.23^c^±0.041	2.06^c^±0.029
	C-18:6	5.25^b^±0.038	2.18^b^±0.021
	I-16:8	5.26^a^±0.036	2.43^a^±0.012
Blue			
	C-23:1	3.71^c^±0.015	0.51^c^±0.064
	C-18:6	3.80^b^±0.012	0.79^b^±0.038
	I-16:8	3.94^a^±0.009	1.27^a^±0.011
White			
	C-23:1	4.30^c^±0.019	1.20^c^±0.049
	C-18:6	4.36^b^±0.016	1.31^b^±0.038
	I-16:8	4.44^a^±0.014	1.43^a^±0.029
p-value		0.004	0.000

Means carrying different superscripts in the same column are significantly different at p≤0.05 or highly significantly different at p<0.01. Means carrying the same superscripts in the same column are non-significantly different at p<0.05. L=Light hours, D=Dark hours, C=Continuous, I=Intermittent, TBC=Total bacterial count, TEC=Total *Enterobacteriaceae* count, SE=Standard error

## Discussion

Modern broiler industry has been focusing on genetically selected broilers for fast growth and rapid WG at the expense of livability, immunity, and leg problems that may be developed. Challenging conditions demand control over the early growth in broilers through conserving feed conversion and livability with a good opportunity for lungs, heart, and skeletal muscles to develop before muscle tissue rapid formation [25]. Light programs have been manipulated in the broiler industry to capture high gain during the grow out. Light sources have been modified over the years from incandescent and fluorescent lamps to LED for their improving influence over growth performance as recorded by Kim *et al*. [26] and Riber [27].

In our study, red and blue LED lights were evaluated against standard medium white LED light. The results have shown that the blue monochromatic LED light enhanced production performance and CW compared to white and red lighting systems. These improvements might be attributed to the calming influence of blue light, and thus directed energy toward WG and FCR compared to red and white lights. Many researchers suggested that using blue light (single or combined) has numerous advantages as Abdel-Azeem and Borham, [28] who agreed with our results while testing the influence of LED red, blue, green, white, and mixed lights and found that blue LED lights with bird density 10/m^2^ were able to keep broilers calmers with synergistic influence on productive performance. Zhang *et al*. [29] declared that monochromatic blue LED light improved LBW and pectoral muscle growth in broilers. In addition, Archer [30] found that blue LED lights emitted cool temperature and were able to improve WG and food conversion ratio, as well as mitigate the impact of stresses and fear. On the contrary, Son and Ravindran [31] and Assaf *et al*. [32] recorded no significant influence on the WG of broilers supplied with three different colors of light (white, blue, or red).

Abdo *et al*. [33] recommended using monochromatic blue light in the summertime for its significant role in modifying heat shock biomarker activities toward enhancing immunity levels and reduce the negative impacts of heat stress. Cao *et al*. [34] also agreed and found a higher growth rate and carcass quality in broilers exposed to blue or green rather than red and white.

The current results revealed a significant improvement in GH, T3, T4, and insulin serum levels in broilers reared in blue compared to white and red LED lights. T3, T4, and insulin usually affect homeostasis; they increased in response to increased GLUCO, TG, and TC in blood. Since broiler activity was limited in blue than in red and white lights, GLUCO, TG, and TC levels were higher, and so the metabolic hormonal levels increased to increase metabolic activity and direct energy toward muscular development. Physiologically, GH is secreted in response to photoperiod with lower body activity, conditions that were available in broilers reared in blue light. The results were supported by those of Zhang *et al*. [35] who studied the influence of white, red, green, and blue LED lights on broiler’s growth. They found GHRH proteins in hypothalamus and plasma GH levels increased in broilers reared in green and blue lights by 6.83-31.36%. Yu *et al*. [36] recorded that T4 and testosterone hormones increased significantly in broilers exposed to the appropriate intensity of green and blue lights. On the contrary, Kumar [37] disagreed with our findings, reporting no significant differences in GLUCO, total protein, blood urea nitrogen, glutathione peroxidase, and superoxide dismutase among broilers exposed to blue, green, and white lights.

Blue LED light in our study improved the titer of anti-ND vaccine antibodies with significant reduction of intestinal bacterial load. The results were supported with those of Li *et al*. [38] who revealed the ability of green and blue lights to improve blood antioxidant (total antioxidant capacity, superoxide dismutase, and glutathione peroxidase) and subsequently increase B-lymphocyte proliferation in bursa of Fabricius in broilers depending on enhanced melatonin levels. Zhang *et al*. [39] agreed with our results and recorded a significant increase in anti-ND antibodies and elevated proliferation of B and T-lymphocytes in broilers exposed to blue monochromatic light by 11.9-40.3% and 10.4-36.2%, respectively. They also recommended a combination of green and blue LED light to enhance the immune function of broilers. Chen *et al*. [40] reported that blue and green lights can induce T-lymphocyte proliferation by 9.57%-32.03% *in* *vivo* and 34.30%-50.53% *in* *vitro* trial. Guo *et al*. [41] agreed with our findings; they found an improvement of a-Naphthyl-acetate esterase and increased antibody production in broilers exposed to intermediate or low-intensity blue lights for the increased melatonin or activation of T and B lymphocyte proliferation.

Circadian rhythm control by the pineal gland is usually affected by the light to dark durations. Our study investigated three lighting regimens; continuous 23L:1D, continuous 18L:6D, and intermittent 16L:8D (4L:2D, 4 times). The results revealed a significant improvement in performance parameters, growth and metabolic hormones, and biochemical and immune profile in broilers reared in continuous 23L:1D, and continuous 18L:6D compared to intermittent 16L:8D. Honda *et al*. [42] tested continuous 12 h white and 12 h blue light and found no disruption in the circadian rhythm of broilers. Lilburn and Loeffler [43] also agreed and stated that continuous lighting regimen is required for the development of digestive organs and WG in broilers. Dereli *et al*. [44] agreed with our findings; they tested 23L:1D or increasing duration of light and light intensity 20 lux (reduced intensity) in 272 male Ross broilers, they found enhanced performance parameters and carcass quality characteristics in broilers exposed to increased photoperiod with reasonable intensity. They also found enhanced resistant against environmental stressors.

Sun *et al*. [45] disagreed with our results, they evaluated 16L:8D, 23L:1D, decreasing-increasing (Dec-Inc), and intermittent 3L:1D and found lower tendency in Dec-Inc regimen for abdominal fat deposition, larger testis, comb percentage, and higher testosterone levels compared to intermittent 3L:1D and 23L:1D regimens. Furthermore, Olanrewaju *et al*. [46] did not agree with our results; they evaluated three photoperiods long-continuous 23L:1D, regular-intermittent 2L:2D, and short-non-intermittent 8L:16D from day 8 to day 56. They found a significant increase (p≤0.05) in LBW, BWG, FI, and CW in broiler reared in short-non-intermittent 8L:16D regimen, suggesting that regular-intermittent 2L:2D regimen can replace long-continuous 23L:1D one in broiler houses to save energy.

## Conclusion

Blue LED light was able to improve significantly productive performance, CW, some biochemical parameters, growth and metabolic hormones, and efficiency of Newcastle vaccine, as well as significantly reduce intestinal bacterial load compared to traditional white and red LED lights. Continuous 18L: 6D hours and 23L: 1D hours regimens at medium accepted intensity prove their efficacy in delivering the influence of blue light on the measured parameters, as well as enhanced growth and metabolism through their direct influence on hypothalamic hormones.

## Authors’ Contributions

ESS designed the experiment, participated, supervised the execution, and participated in writing the manuscript. RAH participated in the execution of the experiment, and in writing the manuscript. All authors read and approved the final manuscript.
